# Impact of habitual chewing on gut motility via microbiota transition

**DOI:** 10.1038/s41598-022-18095-x

**Published:** 2022-08-15

**Authors:** Fukie Yaoita, Keita Watanabe, Ikuo Kimura, Masayuki Miyazawa, Shinobu Tsuchiya, Makoto Kanzaki, Masahiro Tsuchiya, Koichi Tan-No

**Affiliations:** 1 Division of Pharmacology, Faculty of Pharmaceutical Science, Tohoku Medical and Pharmaceutical University, 4-4-1 Komatsushima, Aoba-ku, Sendai, 981-8558 Japan; 2 Department of Applied Biological Science, Graduate School of Agriculture, Tokyo University of Agriculture and Technology, Fuchu, Tokyo 183-8509 Japan; 3 Laboratory of Molecular Neurobiology, Graduate School of Biostudies, Kyoto University, Yoshidakonoe-cho, Sakyo-ku, Kyoto 606-8501 Japan; 4 Department of Orthodontics and Speech Therapy for Craniofacial Anomalies, Tohoku University Hospital, Sendai, 980-8574 Japan; 5 Graduate School of Biomedical Engineering, Tohoku University, 6‑6‑04‑110, Aoba, Aramaki, Aoba‑ku, Sendai, 980‑8579 Japan; 6 Department of Nursing, Tohoku Fukushi University, 1-8-1 Kunimi, Aoba-ku, Sendai, 981-8522 Japan

**Keywords:** Gastrointestinal diseases, Motility disorders, Constipation, Digestive signs and symptoms, Disease prevention, Lifestyle modification, Gastroenterology, Health care

## Abstract

The gut environment, including the microbiota and its metabolites and short-chain fatty acids (SCFA), is essential for health maintenance. It is considered that functional recovery treatment for masticatory dysphagia affects the composition of the gut microbiota, indicating that habitual mastication, depending on the hardness of the food, may affect the gut microbiota and environment. However, the impact of chronic powdered diet feeding on the colonic condition and motility remains unclear. Here, we evaluated various colonic features in mice fed with powdered diets for a long-term and a mouse model with masticatory behavior. We observed a decreased abundance of the SCFA-producing bacterial genera in the ceca of the powdered diet-fed mice. Based on the importance of SCFAs in gut immune homeostasis and motility, interestingly, powdered diet feeding also resulted in constipation-like symptoms due to mild colitis, which were ameliorated by the administration of a neutrophil-depleting agent and neutrophil elastase inhibitors. Lastly, the suppressed colonic motility in the powdered diet-fed mice was significantly improved by loading masticatory activity for 2 h. Thus, feeding habits with appropriate masticatory activity and stimulation may play a key role in providing a favorable gut environment based on interactions between the gut microbiota and host immune system.

## Introduction

The complex synergistic impact of gut microbiota on systemic health has recently been well acknowledged. Unfavorable changes in the composition and function of the gut microbiota may result in host–microbiota interactions followed by an impairment of the host's immune system^1^. Furthermore, alterations in the gut microbiota are directly associated with chronic constipation and inflammatory bowel disease (IBD) with colonic permeability and immune activation^1,2^. Symptoms of these diseases, such as constipation and diarrhea, are associated with changes in the water channel aquaporin, which is used as a target for treatment^3^. Wang et al. reported that blocking aquaporin-4 (AQP-4) may be a novel therapeutic approach for IBD^4^.

The gut microbiota also provides large amounts of short-chain fatty acids (SCFAs), mainly consisting of acetate, propionate, and *n*-butyrate, through the fermentation of dietary fiber^1,5^. SCFAs directly stimulate G-protein-coupled receptors (GPRs), such as GPR41 and GPR43, allowing for various physiological functions, such as regulation of host energy metabolism, gut immune homeostasis, and motility^1,5,6^. Soret et al. reported that SCFAs have an inhibitory effect on enteric neuronal nitric oxide synthase (nNOS)^6^. Alteration of nNOS activity, which catalyzes the formation of nitric oxide (NO) and initiates smooth muscle relaxation, results in gut motility dysfunction^7,8^. Interestingly, an association between a decrease in SCFAs and impaired immune homeostasis involving the infiltration of neutrophils and proinflammatory cytokine expression of macrophages has been observed in the colonic tissue of patients with IBD^1,9,10^.

The maintenance of diets consisting of appropriately hard food has been shown to significantly impact physical and mental health^11–14^. Recovery interventions for masticatory dysphagia caused by strokes have been shown to ameliorate abnormalities in the stool microbiota of patients despite unchanged diets^15,16^. Gut microbiota alterations associated with oral ingestion have been reported to be complex and multifactorial and mastication based on regular diets significantly influences gut condition and function. In addition to food digestion and nutrient absorption, mastication also stimulates motility and fluid secretion in the gut through the cephalic-vagal reflex and humoral system^17^. Based on these observations, mastication of chewing gum has been clinically applied in the postoperative treatment of ileus to facilitate gut motility, improve flatulence, and promote defecation^17–19^. Thus, unfavorable feeding habits, such as soft-food diets, may negatively affect the gut microbiota diversity and SCFA production, although the current evidence is insufficient.

Therefore, the present study aimed to detect impaired colonic motility in terms of the SCFA production associated with the alteration of host–microbiota interactions by identifying the effects of long-term feeding with powdered diets on mice. Furthermore, a unique gnawing model established by our group for prompting mice to specific masticatory behaviors was utilized to observe for beneficial effects of masticatory stimulation in the improvement of impaired colonic function^20,21^.

## Results

### Influence of long-term powdered diet feeding on colonic motility in mice

**Figure 1:**
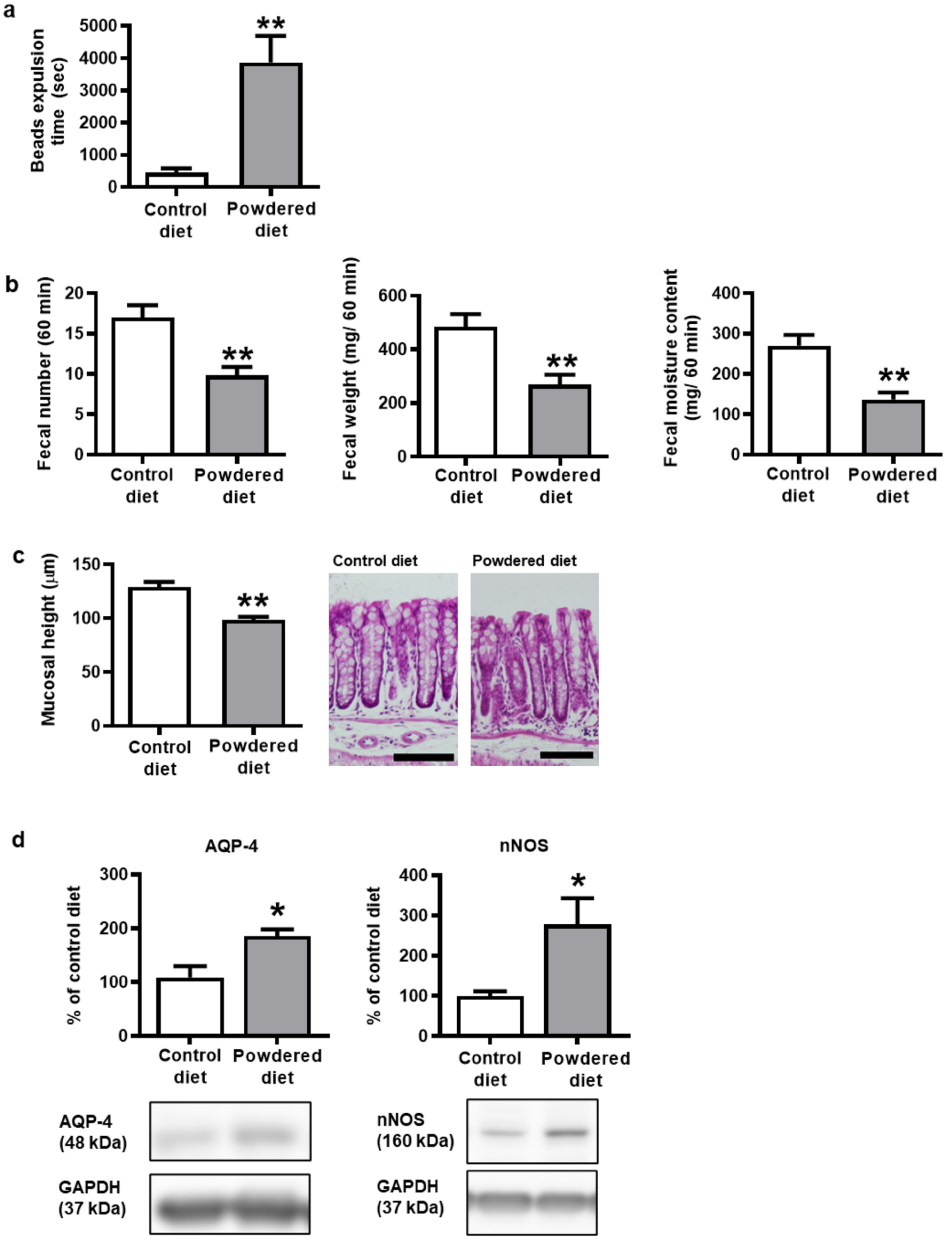
Effects of long-term powdered diet feeding on the colonic motility, colonic mucosal length, and expressions of aquaporin-4 (AQP-4) and neural nitric oxide synthase (nNOS) in the colons of mice. (**a**) Bead expulsion test (p = 0.0024, n = 4 or 5 per group). (**b**) Fecal pellet output, total fecal weight, and fecal water content (p = 0.0011, 0.0019, and 0.0005, respectively, n = 10 per group). (**c**) The lengths of the colonic mucosa (p < 0.0001, n = 5 or 6 per group). (**d**) Western blotting analysis of aquaporin 4 and nNOS (p = 0.0141 and 0.0281, respectively, n = 3–8 per group). Full length blots are presented in Supplementary Fig. S4. The data are given as the mean ± SEM for a group. Student’s *t*-test was used to determine statistical significance. *p < 0.05, **p < 0.01; vs. control diet-fed group.

First, we assessed the effects of a long-term diet consisting of powdered food on colonic motility in mice. Interestingly, the powdered diet-fed group had significantly longer bead expulsion times than the control diet-fed group (Fig. 1a). Moreover, the number of fecal pellets, total fecal weight, and fecal moisture content were significantly decreased in the powdered diet-fed group compared with those in the control diet-fed group (Fig. 1b). Furthermore, morphometric reduction in colonic mucosal length was observed in the powdered diet-fed group (Fig. 1c). Lastly, immunoblotting analysis of the colon tissues of the powdered diet-fed mice also revealed significant increases in protein levels of AQP-4 and nNOS compared with those of the control diet-fed mice (Fig. 1d).

### Influence of long-term powdered diet feeding on cecal levels of SCFAs, colonic expression of GPRs, and cecal microbiota

**Figure 2:**
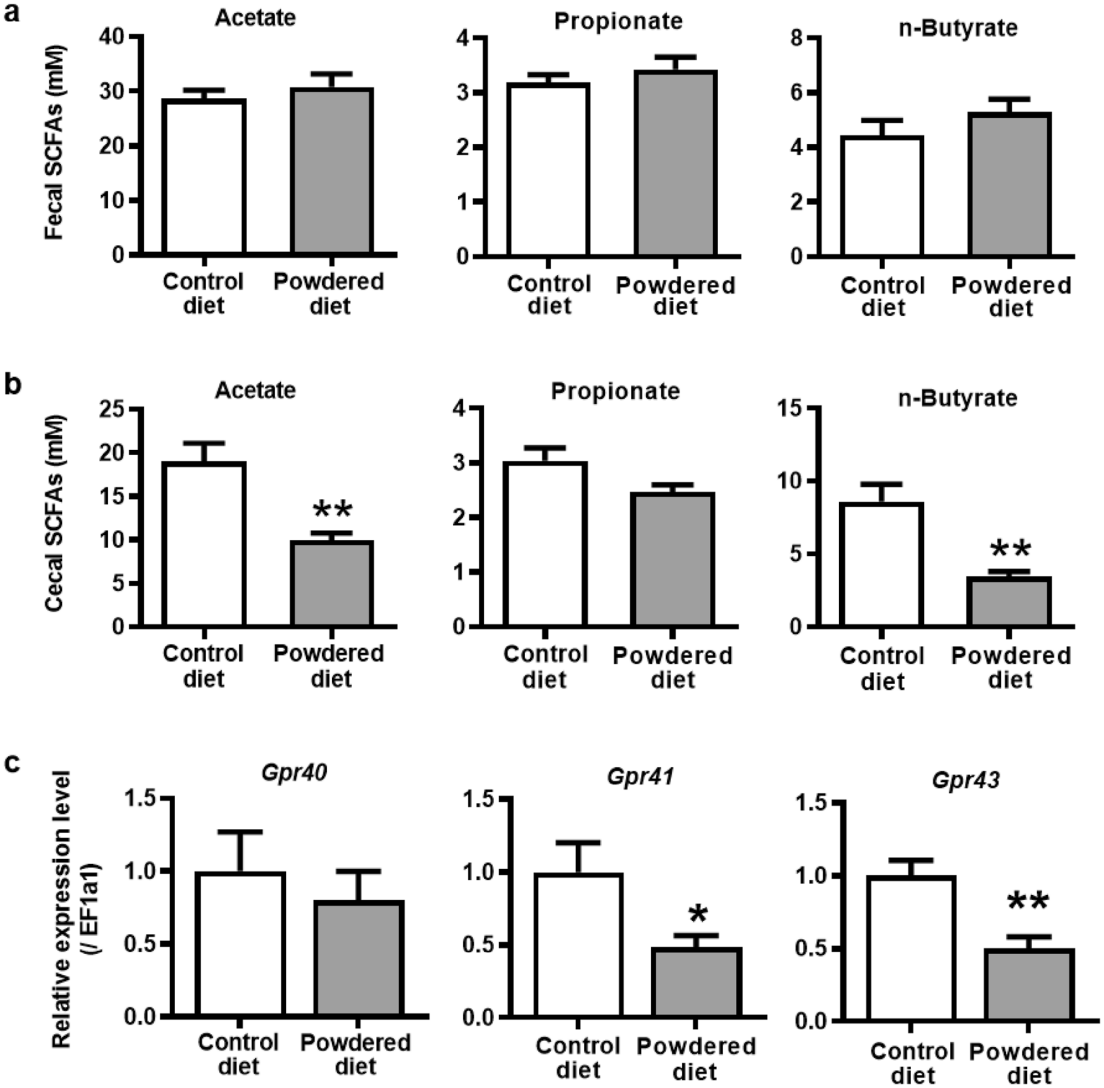
Effects of long-term powdered diet feeding on the short-chain fatty acid (SCFA) levels in (**a**) feces and (**b**) cecal contents, and (**c**) colonic expressions of *Gpr40*, *Gpr41,* and *Gpr43* in mice starved for 5 h. The amounts of acetate, propionate, and n-butyrate among the SCFAs extracted from the samples collected from the feces (p = 0.4519, 0.3856, and 0.2578, respectively, n = 8 per group) and the cecum (p = 0.0013, 0.0506, and 0.0012, respectively, n = 8 per group). Student’s *t*-test was used to determine statistical significance. PCR analysis of *Gpr40*, *Gpr41*, and *Gpr43* in the colons of mice (p = 0.5562, 0.0258, and 0.0011, respectively, n = 12 per group). Student’s *t*-test was used to determine statistical significance. The data are given as the mean ± SEM for each group. *p < 0.05, **p < 0.01; vs. control diet-fed group.

We assessed the effects of a long-term diet consisting of powdered food on SCFAs in the cecal contents, plasma, and feces of mice. The levels of acetate and *n*-butyrate, but not propionate, were significantly lower in the cecal contents of the powdered diet-fed mice than in diet-fed mice (Fig. 2b). It should be noted that there was no significant change in the SCFA levels in the plasma (Supplementary Fig. S1) and feces (Fig. 2a). Reportedly, GPR41 and GPR43 are receptors that are potentially activated by SCFAs, whereas GPR40 is potentially activated by medium- or long-chain fatty acids^22^. As shown in Fig. 2c, long-term feeding with a powdered diet significantly decreased the mRNA expression of *Gpr41* and *Gpr43*, but not that of *Gpr40*, in the colon tissues. Moreover, our preliminary experiment showed that the number of fecal pellets in the mice fed the powdered diet was significantly increased with the administration of sodium butyrate (1200 mg/kg, i.p.) (Supplementary Fig. S2).

**Figure 3:**
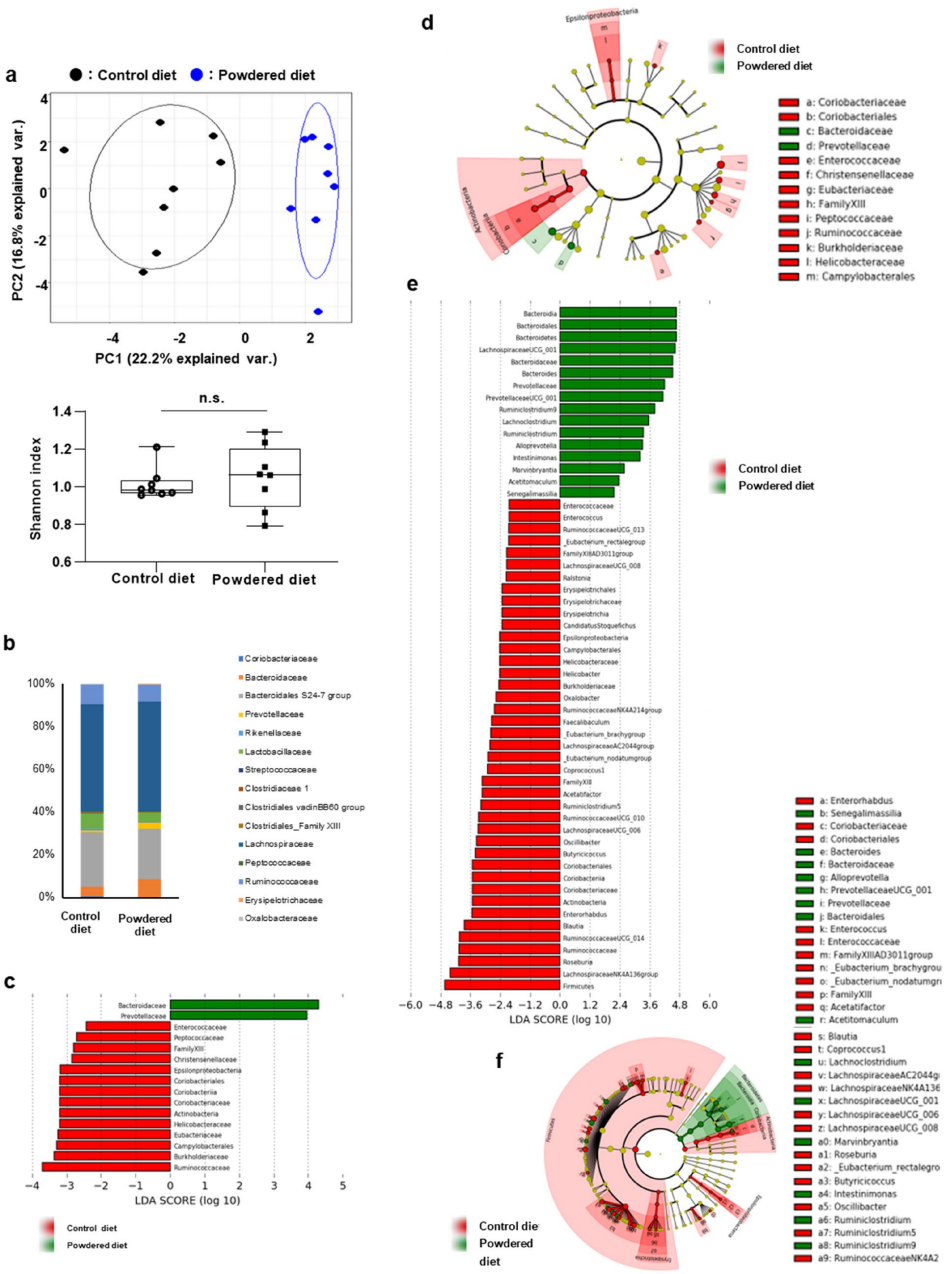
Effects of long-term powdered diet feeding on the cecal microbiota in mice starved for 5 h. (**a**) Cecal microbiota compositions determined as their beta diversity as shown via principal component analysis (PCA) at the family level (p = 0.004). There was no difference between the groups in alpha diversity. PERMANOVA was used to determine statistical significance. (**b**) Relative abundance of major taxonomic groups at the family level. (**c**) Bar chart and (**d**) cladogram of results of linear discriminant analysis (LDA) and effect size (LEfSe) algorithm of fecal microbiota at the family level. (**e**) Bar chart and (**f**) cladogram of results of LDA and LEfSe algorithm of fecal microbiota at the genus level. n = 8 per group. (**d**) and (**f**) were generated using the Huttenhower lab's Galaxy module (http://huttenhower.sph.harvard.edu/galaxy/) for LEfSe v1.0102.

As the SCFA levels in the cecal contents were decreased in the powdered diet-fed mice and the influence of the cecal microbiome on the colon has been suggested^23^, the cecal microbiota composition was confirmed here, as indicated by the principal component analysis (PCA) and alpha diversity based on taxonomic datasets (Fig. 3). Although there was no significant difference in the alpha diversity, the PCA of taxonomic groups at the family levels of cecal microbiota showed significant segregation between the powdered diet-fed and control diet-fed groups in the beta diversity. A pairwise comparison using the permutational multivariate analysis of variance (PERMANOVA) revealed that the differences between the groups were statistically significant (p = 0.004, Fig. 3a). A taxonomic analysis of the cecal microbiota at the family level showed no significant changes that reflected the results of the PCA (Fig. 3b).

**Figure 4:**
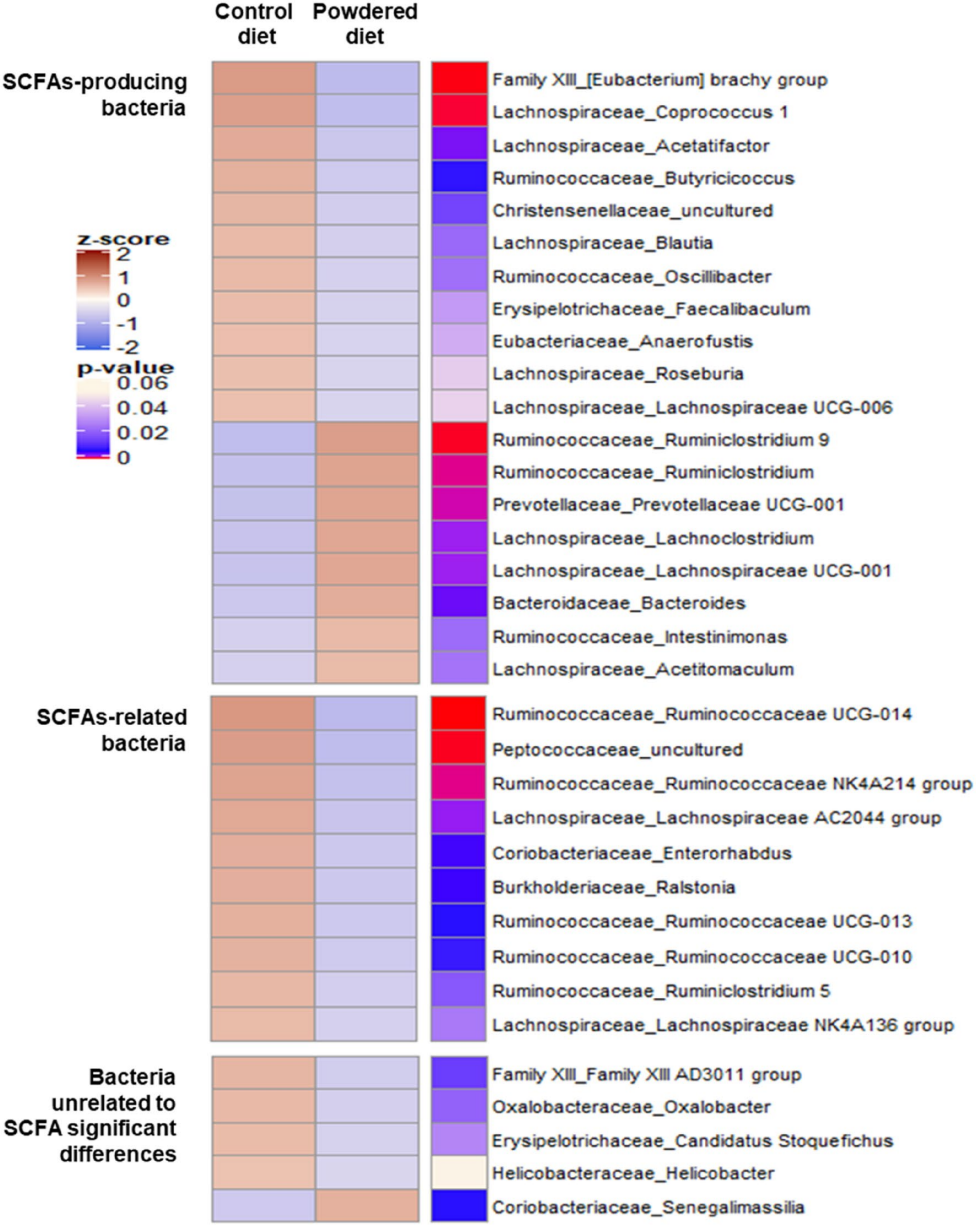
Heat map of relative abundance of taxa with significant differences at the genus level for SCFAs-producing bacteria, SCFAs-related bacteria (bacteria that grow on SCFAs, bacteria whose presences are directly proportional to the level of SCFAs, or close relatives of SCFA-producing bacteria), and bacteria unrelated to SCFAs, in the cecal microbiota compositions comparing long-term powdered diet-fed mice and control diet-fed mice (mean relative abundance > 0.1%). The abundance of each genus is plotted in a brown–blue color scale. The brown and blue colors indicate high and low abundance, respectively. n = 8 per group.

Next, the linear discriminant analysis (LDA) effect size (LEfSe) algorithm was applied to further identify the taxa at the family and genus levels between the control and powdered diet-fed mice. A bar chart (Fig. 3c) and cladogram (Fig. 3d) were generated from LEfSe confirmed that the bacteria inhabiting the control and powdered diet-fed mice clustered separately. In particular, at the family level, the microbiota associated with the powdered diet was characterized by a predominant increase in Bacteroidaceae and Prevotellaceae members. These bacterial family members are known to produce SCFAs. Therefore, these changes did not reflect lower SCFA levels in the cecal contents of the powdered diet-fed group. In addition, Fig. 3e,f shows the results at the genus level. From these results, bacterial genera with a significant difference between the control and powdered diet-fed groups were selected and displayed using a heatmap (Fig. 4). These bacterial genera were divided into the following three areas. The upper, middle, and bottom areas of the heatmap categorized the SCFA-producing bacteria, SCFA-related bacteria (bacteria that grow on SCFAs, bacteria whose presence are directly proportional to the level of SCFAs, or close relatives of SCFA-producing bacteria), and bacteria unrelated to SCFAs, respectively. As shown in the upper area of the heatmap, indicating the SCFA-producing bacteria, the number of bacterial genera in the cecum of the powdered diet-fed mice was less than that of the control diet-fed mice, although some SCFA-producing bacteria were more abundant in the powdered diet-fed group. Moreover, in the middle area of the heatmap, indicating the SCFA-related bacteria, the bacterial genera were not abundant in the cecum of the powdered diet-fed mice. These results collectively indicate that long-term powdered diet feeding modifies the cecal microbiome with a relatively large number of SCFA-producing and -related genera.

### Involvement of neutrophils rather than macrophages in the colonic motility and inflammation in long-term powdered diet-fed mice

**Figure 5:**
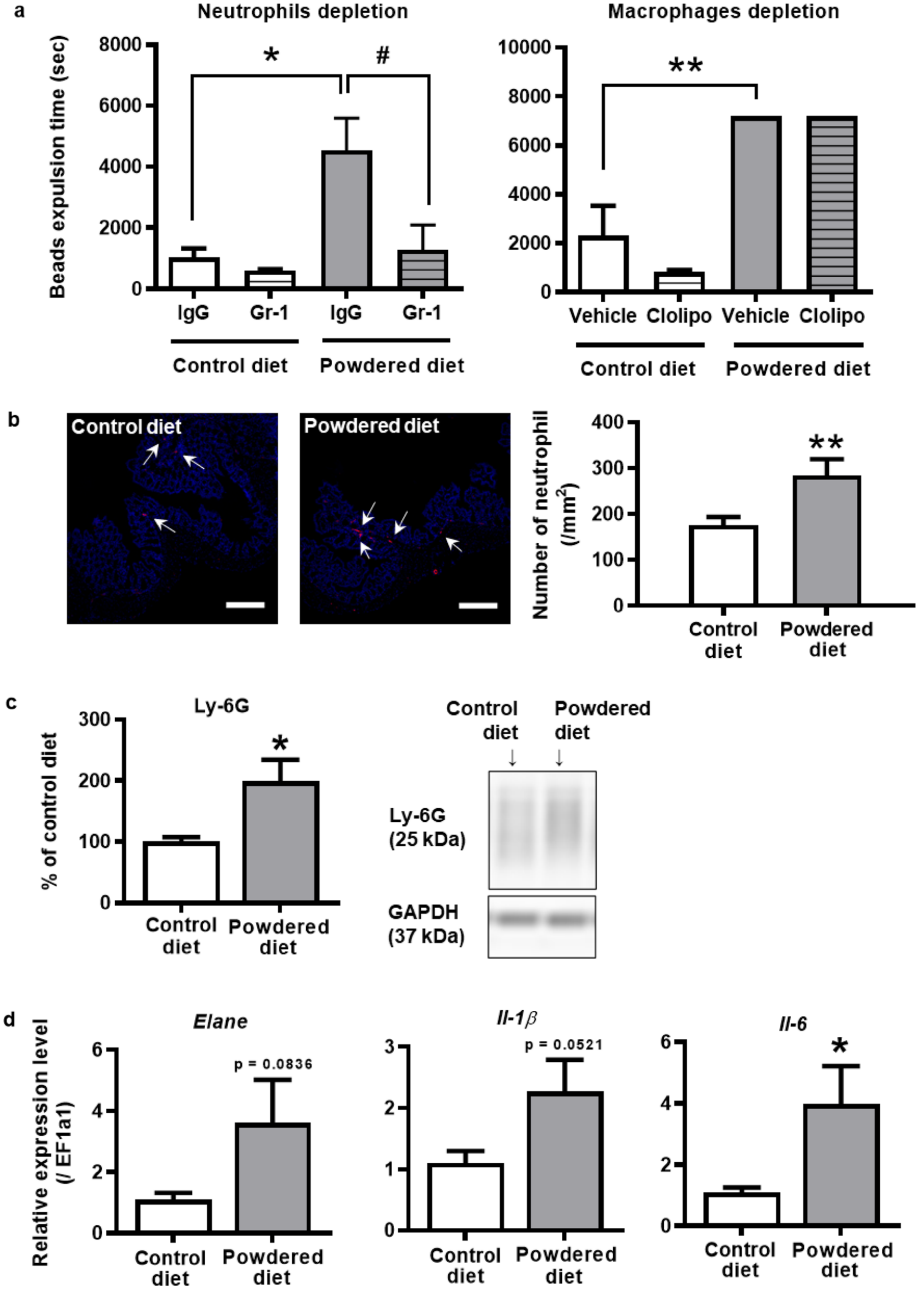
Involvement of immune cells in the constipation-like symptoms of the long-term powdered diet-fed mice. (**a**) Colonic motility of mice with macrophage or neutrophil depletion in the bead expulsion test (n = 3–5 per group). In the left panel, one-way analysis of variance (ANOVA): F (3, 14) = 6.024, p = 0.0075, with Tukey's test. In the right panel, one-way ANOVA: F (3, 8) = 29.82, p = 0.0001, with Tukey's test. *p < 0.05, **p < 0.01; vs. vehicle-treated control diet-fed group. ^#^p < 0.05; vs. vehicle-treated powdered diet-fed mice group. (**b**) Recruitment of neutrophils in the colons of mice. Representative images showing colons stained with antibodies against Gr-1 (red) and DAPI (blue) (p = 0.0067, n = 6–7 per group). Student’s *t*-test was used to determine the statistical significance. **p < 0.01; vs. control diet-fed group. (**c**) Western blotting analysis of Ly-6G in the colons of mice (p = 0.0152, n = 8 per group). Student’s *t*-test was used to determine statistical significance. *p < 0.05; vs. control diet-fed group. Full length blots are presented in Supplementary Fig. S5. (**d**) PCR analysis of *Elane*, *IL-1β* and *IL-6* in the colons of mice (p = 0.0836, 0.0521, and 0.0308, respectively, n = 5–6 per group). Student’s *t*-test was used to determine statistical significance. *p < 0.05; vs. control diet-fed group. The data are given as the mean ± SEM for each group.

We examined the involvement of immune cells such as neutrophils and macrophages in the impairment of colonic motility. To discern the effects of neutrophils or macrophages, we administered depleting agents: anti-Gr-1 Ab for neutrophils and clodronate liposomes for macrophages. The delayed bead expulsion time in the powdered diet-fed mice was significantly suppressed in the anti-Gr-1-treated mice compared to that in the control IgG-treated mice (Fig. 5a, left panel). However, there were no significant changes in the expulsion time between the clodronate-liposome- and vehicle (negative control for clodronate-liposome)-treated powdered diet-fed mice (Fig. 5a, right panel). Additionally, there was no significant effect on the depletion in the control diet-fed mice (Fig. 5a).

Next, to further explore the involvement of neutrophils in the reduced colonic motility caused by long-term powdered diet feeding, the expression of Ly-6G was examined using immunohistochemistry or western blotting. These results showed that the neutrophils were significantly increased in the colons of the powdered diet-fed mice compared with those of the control diet-fed mice (Fig. 5b,c). Moreover, the expression of neutrophil elastase (*Elane*), *IL-1β*, and *IL-6*, which are associated with neutrophil activity, were examined using polymerase chain reaction (PCR). As shown in Fig. 5d, *IL-6* expression was significantly upregulated in the colon tissues of the powdered diet-fed mice compared with that of the control diet-fed mice. Similarly, the expression of *Elane* and *IL-1β* displayed increasing trends that were not significant (*Elane*, p = 0.08; *IL-1β*, p = 0.05).

**Figure 6:**
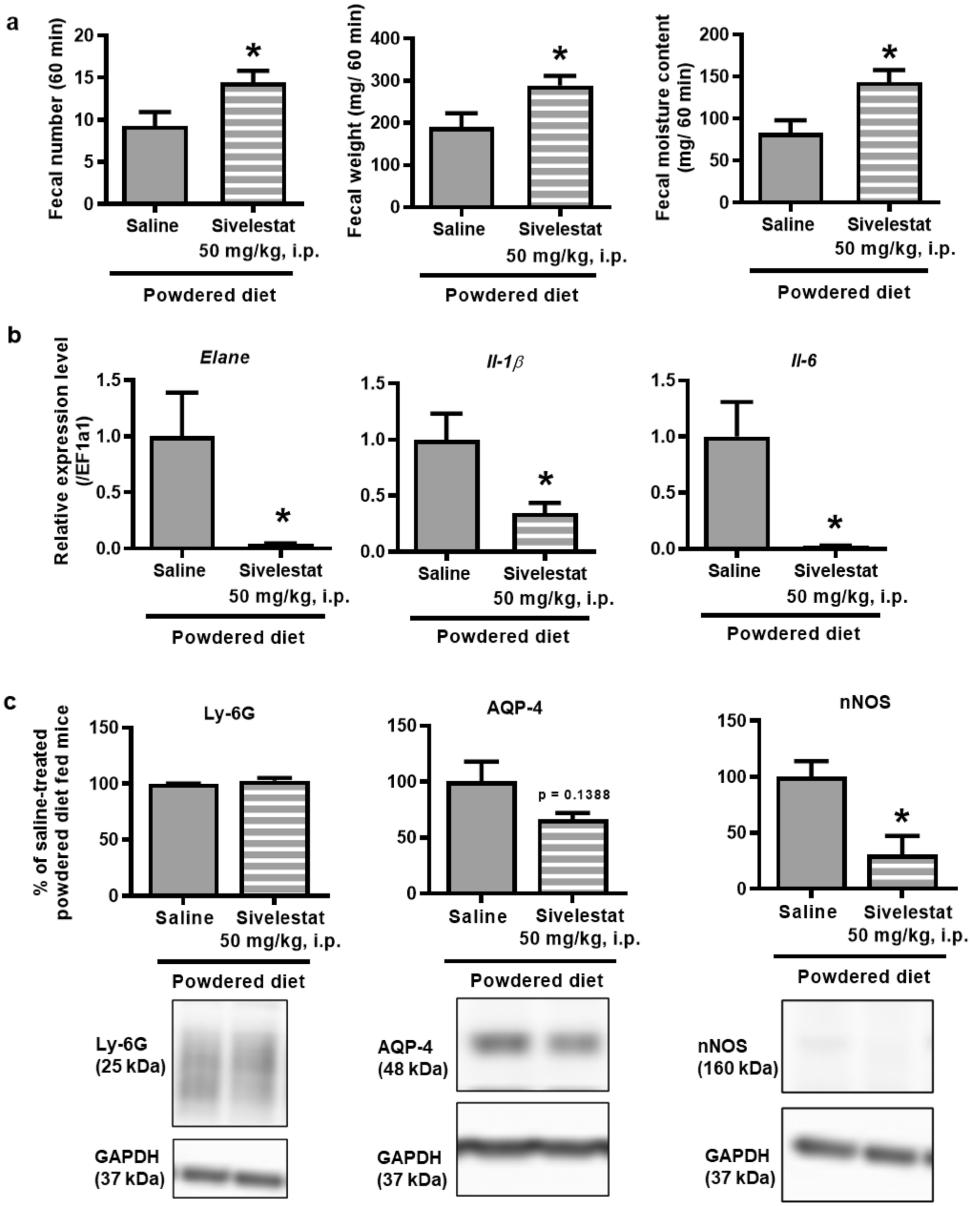
Effect of sivelestat (50 mg/kg, i.p.), a neutrophil elastase inhibitor, on the constipation-like symptoms of long-term powdered diet-fed mice. (**a**) Fecal pellet count, total fecal weight, and fecal moisture content (p = 0.0344, 0.0314, and 0.0140, respectively, n = 7 per group), (**b**) PCR analysis of *Elane*, *IL-1β* and *IL-6* in the colons of mice (p = 0.0388, 0.0300, and 0.0136, respectively, n = 5 per group), and (**c**) western blotting analysis of Ly-6G, aquaporin-4 (AQP-4), and neural nitric oxide synthase (nNOS) in the colons of the mice (p = 0.3055, 0.1388, and 0.0399, respectively, n = 3 per group). Full length blots are presented in Supplementary Fig. S6. The data are given as the mean ± SEM for a group. Student’s *t*-test was used to determine statistical significance. *p < 0.05; vs. vehicle-treated powdered diet-fed mice group.

In addition, the *Elane* inhibitor, sivelestat, effectively increased the number of fecal pellets, total fecal weight, and fecal moisture content in the long-term powdered diet-fed mice (Fig. 6a). However, there was no effect on these indicators with the inhibitor administration in mice fed the control diet (Supplementary Fig. S3). In terms of neutrophil-related gene expression, sivelestat administration significantly decreased the expression of *Elane*, *IL-1β*, and *IL-6* in the colon tissues of powdered diet-fed mice compared with that of the vehicle-treated powdered diet-fed mice. Moreover, the level of nNOS, but not that of Ly-6G and AQP-4, in the colon tissues of powdered diet-fed mice also decreased after sivelestat administration (Fig. 6b,c).

### Beneficial effects of 2-h masticatory activity on delayed colonic motility induced by long-term powdered diet feeding

**Figure 7:**
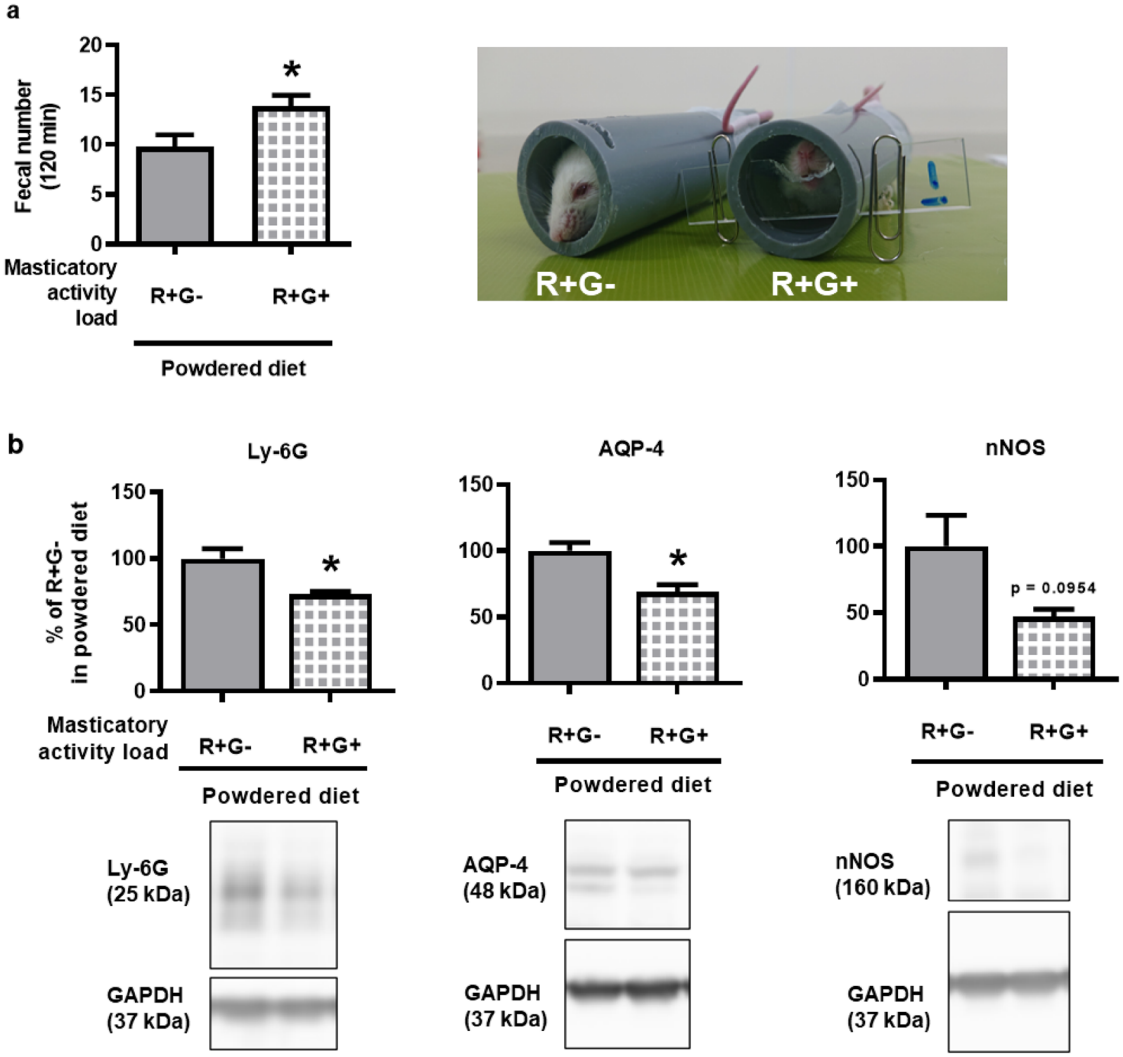
Effect of 2-h masticatory activity on the constipation-like symptoms of long-term powdered diet-fed mice. (**a**) Fecal pellet output (p = 0.0214, n = 13 per group) and (**b**) western blotting analysis of Ly-6G, aquaporin 4, and neural nitric oxide synthase (nNOS) expression (p = 0.0234, 0.0201, and 0.0954, respectively, n = 3 per group) in the colons of long-term powdered diet-fed mice subjected to the 2-h restraint-gnawing (R + G +) protocol. Full length blots are presented in Supplementary Fig. S7. The data are given as the mean ± SEM for a group. Student’s *t*-test was used to determine statistical significance. *p < 0.05; vs. R + G− group.

To investigate the mechanisms behind masticatory behavior preventing delayed colonic motility, we employed our mastication-like behavior (gnawing) model (2 h) and measured its effect on colonic motility after long-term feeding with a powdered diet^21^. As shown in Fig. 7a, the number of fecal pellets was significantly higher in the R + G + group than in the R + G− group. Indeed, as shown in Fig. 7b, the level of Ly-6G and AQP-4 was significantly lower in the colon tissues of the R + G + group than in the R + G− group among the powdered diet-fed mice. Furthermore, the nNOS levels showed a decreasing trend that was not statistically significant (p = 0.10). Interestingly, it should be noted that the beneficial effects of masticatory behavior on colonic motility were statistically robust in the control diet-fed groups. These R + G + and R + G- groups were 12.3 ± 1.01 and 17.0 ± 1.02, respectively, with a significant difference of 0.0039.

## Discussion

The present study was designed to investigate the potential harmful effects of unfavorable feeding habits, such as soft food diets, focusing on how they affect colonic motility by altering gut microbiota diversity and local production of SCFAs. Our key finding was that mild colitis with neutrophil recruitment caused by powdered diet feeding resulted in constipation-like symptoms associated with decreased levels of SCFAs due to shifts in the gut microbiota. Furthermore, masticatory behavior was found to improve the constipation-like and inflammatory symptoms in the powdered diet-fed mice as seen in the observed improvement in colonic neutrophil recruitment. Consistent with this notion, masticatory behavior has been acknowledged as a potential management option for patients recovering from ileus by facilitating gut motility^17–19^.

In this study, we first investigated the effects of a powdered diet on the colon. We found that it induced delays in colonic motility, decreased colonic mucosal thickness, and caused mild colitis as indicated by increased neutrophil recruitment and cytokine expression. Interestingly, we found that different diet properties, with the same nutritional components, caused changes in the cecal microbiota composition. This microbiota alteration was determined by the decreased proportion of SCFA-producing and SCFA-related bacteria, leading to the decreases in levels of acetate and n-butyrate in the cecal contents, but not in the plasma or feces. These findings may suggest that the changes in SCFAs were localized to the cecum. Moreover, in long-term powdered diet-fed mice, the fecal moisture content and weight were low, and the contents remained in the colon for a long time (Fig. 1a,b). Therefore, although there were low levels of SCFA in the cecal contents of powdered diet-fed group, it was speculated that no significant difference in plasma or feces between the control and powdered diet-fed groups was detected.

It is well known that SCFAs stimulate GPRs to regulate gut motility and inhibit the effects of butyrate on nNOS and colonic epithelial cell proliferation^1,5,6,24^. These findings support our hypothesis that the decreased levels of butyrate in the cecal contents of the powdered diet-fed mice contribute to the suppression of colonic motility and decrease in colonic mucosa thickness. Furthermore, increased expression of AQP-4 in the colons of the powdered diet-fed mice may contribute to fecal moisture loss, which possibly plays a role in developing constipation-like symptoms^3,4,25^.

In addition to regulating colonic function, gut SCFA levels have direct anti-inflammatory effects that substantially modulate neutrophil and macrophage functions in the innate immunity^1,5^. Although the contribution of macrophages in mild colitis remains unknown, the improvement of delayed colonic motility by employing neutrophil depletion and inhibition indicated that neutrophil recruitment with *Elane* expression plays a key role in the development of constipation-like symptoms and chronic mild inflammation associated with long-term powdered diet feeding^9,10^. Interestingly, in terms of GPR expression in intestinal epithelial cells, Kamp et al. reported that the administration of acetate suppressed neutrophil migration via effects on GPR43^26^. Thus, due to the changes in both SCFA levels and GPR expression in the colon of the powdered diet-fed mice, the decrease in GPR stimulation by acetate and butyrate might have directly triggered the recruitment and activation of neutrophils, leading to delayed colonic motility and mild colitis. Moreover, exogenous administration of sodium butyrate in our preliminary experiments significantly improved defecation in the powdered diet-fed mice (Supplementary Fig. S2), indicating that SCFA imbalance in the gut might have increased the risk of the constipation-like symptoms of chronic mild colitis in the long-term powdered diet-fed mice. Therefore, further studies are warranted to identify the mechanisms underlying the alterations in SCFA production of the gut microbiota caused by specific food properties.

Additional 2-h masticatory activity improved the constipation-like symptoms in the long-term powdered diet-fed mice, although swallowing of the gnawed strip component (plastic particles) by mouse during loading is inevitable. In addition, in mice, orally ingested indigestible substances have been shown to take more than 3 h to reach the colon^27^. Therefore, in this study, it is unlikely that the particles had an effect on motility during the 2-h masticatory activity load. Furthermore, the masticatory activity decreased the expression of Ly-6G, indicating that habitual masticatory stimulation may attenuate mild colitis by suppressing neutrophil recruitment. The immune response of neutrophils has been reported to be altered by exercise^28–30^, which may be related to the suppression of neutrophil recruitment in the colon by masticatory stimulation. In addition, the decrease in the AQP-4 and nNOS levels in the colon tissues after the masticatory activity improved the attenuation of water reabsorption and colonic motility^3,7,8,25^. These results were consistent with previous studies that acknowledge that chewing gum improves postoperative recovery in patients with ileus via cephalic vagal stimulation^19^. Meanwhile, our results suggest that the issues with masticatory function and habitual feeding, such as a soft diet, may influence daily gut functions and symptoms.

In summary, our findings showed the role of masticatory behaviors in our dietary habits, as seen with the long-term powdered diet feeding inducing constipation-like symptoms and chronic mild colitis involving neutrophil recruitment and activation. The impairment of colonic condition and function may be associated with decreased SCFA levels due to shifts in gut microbiota shift that are caused by long-term feeding with a powdered diet. Thus, our results show the importance of daily masticatory behavior in maintaining colonic condition and function.

## Methods

### Animal treatment

Male BALB/c mice (3 weeks old, 130 in total) were purchased from Japan CREA (Tokyo, Japan) and housed under a strict 12 h light/dark cycle under controlled atmospheric conditions (23 ± 1 °C, 55 ± 5% humidity). The randomly selected mice were fed either a control diet (pellet-type Labo MR stock; Nihon Nosan, Kanagawa, Japan) or a powdered diet (powder-type Labo MR stock; Nihon Nosan) containing the same ingredients with given free access to water for 4 months. A blinded analysis was done to avoid experimental bias. All animal use protocols were conducted in accordance with the Standards of Humane Care and Use of Laboratory Animals of Tohoku Medical and Pharmaceutical University. All animal experiments were approved by the Tohoku Medical and Pharmaceutical University Animal Experiment Committee (Ethic ID No. 20062-cn). All experiments complied with the Guidelines for Care and Use of Laboratory Animals issued by Tohoku Medical and Pharmaceutical University and the ARRIVE guidelines.

### Colonic bead expulsion test

Colonic motility was assessed by measuring the time to the expulsion of a 3 mm diameter glass bead. Prior to testing, the mice were starved for 20 h with water made available ad libitum. The mice were then placed in plastic observation chambers with metal meshes at the bottom. After 30 min, the beads were inserted into the colons 2.5 cm from the anus using a 2-mm diameter silicone tube. The times spent expelling the beads were measured for up to 120 min.

### Selective suppression of neutrophil and macrophage activities

Anti-granulocyte-differentiation antigen-1 antibody (anti-Gr-1 Ab; RB6-8C5) or vehicle (normal rat IgG; Jackson Laboratories, Bar Harbor, ME, USA) was intravenously administered to the mice 24 h before the experiments at doses of 1 mg/kg body weight^30^. Sivelestat (50 mg/kg; Sigma-Aldrich, St. Louis, MO, USA), a neutrophil elastase inhibitor, was administered intraperitoneally 2 h before the collection of feces and colon tissue^31^. Liposomes encapsulating clodronate (clodronate-liposome; HYGIEIA BIOSCIENCE, Osaka, Japan) or vehicle (negative control for clodronate-liposome, HYGIEIA BIOSCIENCE) was intravenously administered to the mice 24 h before the experiments at doses of 100 mg/kg body weight.

### Fecal pellet output, total fecal weight, and fecal moisture content

Non-starved mice were placed in a plastic chamber, and the fecal pellets excreted per unit of time were counted. The fecal moisture content was calculated as the difference between their wet and dry weights.

### Masticatory behavioral model

To examine the effect of masticatory activity on colonic motility, a behavioral experiment adopted by Ayada et al. was used^20^. Briefly, the mice were restrained for 2 h in a cylinder with an inserted plastic plate (thickness 0.1 cm), causing them to gnaw at the strip to escape (“R + G+”) (masticatory activity load group). For the control group, the tails of mice were fixed to a tube using tape (“R + G−”) (non-load group).

### Histological analyses of colon tissues

Portions of the mid colon tissues were washed with PBS, sampled, fixed in 4% paraformaldehyde-PBS, and used for histological assessment. The lengths of the colonic mucosa were measured via hematoxylin–eosin staining of the cryosections, and the number of neutrophils was counted via immunostaining using anti-Gr-1 Ab as previously described^30^. Quantitative analyses were performed using ImageJ software (NIH, Bethesda, MD, USA). The slides were examined under a Biorevo BZ9000 microscope (Keyence, Osaka, Japan).

### Quantitation of SCFAs

SCFAs were prepared as previously described^22,32^. The SCFA levels in the cecal, plasma, and fecal samples of mice starved for 5 h were suspended in water (10 w/v), and then separated by centrifugation (8000*g*, 5 min, 4 °C). Fecal supernatants (350 µL) or plasma (80 µL) samples were immediately mixed with 5-sulfosalicylic acid (2% vol), and vortexed for 1 min. The samples were centrifuged at 15,000*g* for 15 min, and the supernatant was collected. The supernatant was mixed with 2-ethylbutyric acid (30 pmol; internal control), hydrochloric acid (5% vol), and diethyl ether (200% vol), and vortexed for 1 min. The samples were centrifuged at 3000*g* for 5 min, and the SCFA-containing ether layers were collected and quantified using gas chromatography-mass spectrometry (GC–MS) with GCMS-QP2010 Ultra (Shimadzu Corporation, Kyoto, Japan). A VF-WAXms (30 m × 0.25 mm, internal diameter × 1 µm; Agilent Technologies, Santa Clara, CA, USA) was used for chromatographic separation. The mass spectrometer was set to scan mode from *m/z* 60, 74, and 60 (retention times: 9.6, 10.7, and 11.8 min) for acetate, propionate, and n-butyrate, respectively.

### Analysis of gut microbiota using 16S rRNA gene sequencing

Fecal DNA was extracted from frozen samples using the FastDNA SPIN Kits for Feces (MP Biomedicals, Santa Ana, CA, USA) according to the manufacturer’s instructions.

The V3–V4 region of the 16S rRNA gene was amplified using the dual-indexed 341F/806R primers. Amplicons were sequenced using an Illumina MiSeq with a MiSeq Reagent Kit V3 (Illumina, San Diego, CA, USA). The libraries were prepared following the Illumina protocol for 1 pM libraries: “Preparing Libraries for Sequencing on the MiSeq'' (part 15039740, Rev. D). Paired-end sequencing (2 × 301 bp) was performed using the Illumina MiSeq platform. Processing and quality filtering of reads was performed using Quantitative Insights into Microbial Ecology (QIIME, v1.9.1, http://www.qiime.org). Operational taxonomical units (OTU) picking was performed using the open-reference method, which encompasses clustering of reads against a reference sequence collection. To eliminate erroneous mislabeling, the resulting OTU tables were checked for mislabeling sequences. In addition, the chimera-free sequences were aligned with the SILVA database (http://www.arb-silva.de) at 97% identity. Representative raw sequences were further aligned using Python Nearest Alignment Space Termination with the SILVA core-set alignment template (Silva version 128) to obtain bacterial OTU^33^. Diversity analyses were performed using the QIIME script core_diversity_analyses.py. The statistical significance of sample groupings was assessed using permutational multivariate analysis of variance (QIIME script compare_categories.py). The phylogenetic tree was constructed using the FastTree software method in QIIME. Sequence counts for each bacterial OTU and the phylogenetic tree were imported from Qiime into R (× 64, 4.2.0) for PCA with the R package Vegan 2.6-2 and for creating the heatmap of taxonomic relative abundance with the ComplexHeatmap package. LEfSe results were visualized using a taxonomic histogram and cladogram, as implemented on http://huttenhower.sph.harvard.edu/galaxy/ with α = 0.05, the threshold of LDA score at 2.0 and relative abundance greater than 0.1%. The raw data have been deposited at the DNA Data Bank of Japan (DDBJ, https://www.ddbj.nig.ac.jp) database under accession no. DRA012037.

### Quantitative PCR, western blotting, and immunohistochemistry

The animals were sacrificed by decapitation without anesthesia, and their colon tissues were quickly dissected^34^. Total RNAs were analyzed using the BioRad CFX96 system (Bio-Rad, Hercules, CA, USA). The relative expression of target genes was determined using the 2–DCT method and function of elongation factor 1a1 (EF1a1) expression^35^. Western blotting was performed following standard procedures^34^. The antibodies and primers used in this study are shown in Supplementary Tables S1 and S2.

### Statistical analysis

Data are expressed as means ± SEM; they were statistically analyzed using GraphPad Prism version 8 software (GraphPad Software Inc., La Jolla, CA, USA). The statistical significance of differences between means was evaluated using Student’s unpaired *t*-test and one-way analysis of variance (ANOVA), followed by Tukey's test for multiple comparisons. PERMANOVA was used to analyze the similarity of microbiomes. The alpha diversity of each group was measured using Shannon diversity. The FDR q-values in 16S rDNA sequencing were analyzed. The false discovery rate (FDR; q-value) was estimated using Benjamini–Hochberg procedure. The 16S rDNA sequencing data were analyzed using unpaired Student’s *t*-test with FDR correction. Differences were considered statistically significant at p < 0.05 and q < 0.05.

## Supplementary Information

Supplementary Information.

## Data Availability

The datasets of this study are available from the corresponding author on reasonable request. Raw sequence data have been deposited at the DNA Data Bank of Japan (DDBJ, https://www.ddbj.nig.ac.jp) database under accession no. DRA012037.
