# Supplementary material for: Impact of habitual chewing on gut motility via microbiota transition
Source: Sci Rep. 2022 Aug 15;12:13819. doi: 10.1038/s41598-022-18095-x (PMC9378666; doi:10.1038/s41598-022-18095-x)

Supplementary Information

Impact of habitual chewing on gut motility via microbiota transition

F. Yaoita [1*], K. Watanabe [2, 3], I. Kimura [2, 3], M. Miyazawa [1], S. Tsuchiya [4], M, Kanzaki [5], M. Tsuchiya [6*], and K. Tan-No [1]

[1]Division of Pharmacology, Faculty of Pharmaceutical Science, Tohoku Medical and Pharmaceutical University; [2]Department of Applied Biological Science, Graduate School of Agriculture, Tokyo University of Agriculture and Technology; [3]Laboratory of Molecular Neurobiology, Graduate School of Biostudies, Kyoto University; [4]Department of Orthodontics and Speech Therapy for Craniofacial Anomalies, Tohoku University Hospital; [5]Graduate School of Biomedical Engineering, Tohoku University; [6]Department of Nursing, Tohoku Fukushi University

Materials and Methods

Supplementary Table S1. Primers used in this study.

Gene	Forward	Reverse
Gpr40	CAT CCG AGG CGC AGT GTC C	AGG GGC CAG GCT CCA GAA G
Gpr41	GCC GGC GCA AGA GGA TAA	CCA GGG GGT CGA TAC AAG AGT T
Gpr43	GCT GAG GGC GGG CAA CA	CCG GCA TAA CAG TGG AGA CAA GA
Elane	GAG CGC ACT CGA CAG ACC TT	ATG GTA GCG GAG CCA TTG AG
Il-1β	GCA CCT TCT TTT CCT TCA TCT TTG	GTT GTT CAT CTC GGA GCC TGT
Il-6	AAC CAC GGC CTT CCC TAC TT	CCA TTG CAC AAC TCT TTT CTC ATT
Ef1a1	ATT CCG GCA AGT CCA CCA CAA	CAT CTC AGC AGC CTC CTT CTC AAA C

Supplementary Table S2. Antibodies used in this study.

Antibody	Source
Anti-Aquaporin 4 antibody, rabbit polyclonal antibody	Abcam
Anti-nNOS antibody, rabbit polyclonal antibody	Thermo Fisher Scientific
Anti-Ly6G antibody (clone 1A8), rat monoclonal antibody	BioLegend
Anti-GAPDH (D16H11) antibody, rabbit polyclonal antibody	Cell Signaling
Anti-rabbit IgG, HRP-linked antibody	Cell Signaling
Anti-mouse IgG, HRP-linked antibody	GE Healthcare
Normal rat IgG	Wako
Anti-granulocyte-differentiation antigen 1 (Gr-1) antibody (RB6-8C5, rat IgG2b)	Purified from the culture supernatants of hybridoma (provided from Dr. R. Coffman).

Results

Supplementary Figure S1 Information

The following experiments were performed to investigate the influence of long-term powdered diet-feeding on the levels of acetate, propionate, and n-butyrate in the plasma. These methods have been explained in the text. There were no significant changes in SCFA levels in the plasma between the powdered diet- and control diet-fed groups (Supplementary Figure S1).

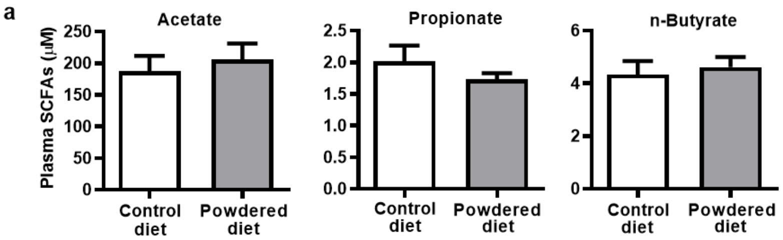

Supplementary Figure S1. Effects of long-term powdered diet feeding on short-chain fatty acid (SCFA) levels in the plasma of mice fasted for 5 h. The levels of acetate, propionate, and n-butyrate among SCFAs extracted from the samples collected from the plasma (plasma; p = 0.6025, 0.2936, and 0.6785, respectively, n = 8/group). Student's t-*t*est. Data are presented as mean ± SEM for each group.

Supplementary Figure S2 Information

The following experiments were performed to investigate the effect of sodium butyrate on constipation-like symptoms in the powdered diet-fed mice. Immediately after intraperitoneal administration of sodium butyrate (1200 mg/kg, i.p.) or saline to non-fasted mice, they were individually placed in plastic chambers. Two hours later, their feces were counted and collected. The fecal moisture content was calculated as the difference between wet and dry weights of the feces. Administration of butyrate significantly reduced the number of fecal pellets, but not total fecal weight and fecal moisture content, in the long-term powdered diet-fed mice (Supplementary Figure 2).

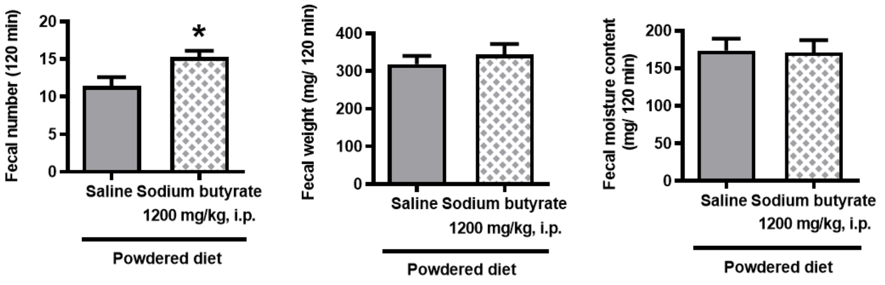

Supplementary Figure S2. Effect of sodium butyrate (1200 mg/kg, i.p.) on the number of fecal pellets, total fecal weight, and fecal water content in the long-term powdered diet-fed mice (p = 0.0176, 0.5569, and 0.9159, respectively, n = 5–10/group). Student's *t*-test. Data are presented as mean ± SEM for each group. *p < 0.05; saline-treated powdered diet-fed mice group.

Supplementary Figure S3. Information

The following experiments were performed to investigate the effect of sivelestat on the number of fecal pellets, total fecal weight, and fecal moisture content in the control diet-fed mice. These methods have been explained in the text. There were no significant differences in the number of

fecal pellets, total fecal weight, and fecal moisture content in the control diet-fed group (Supplementary Figure S3).

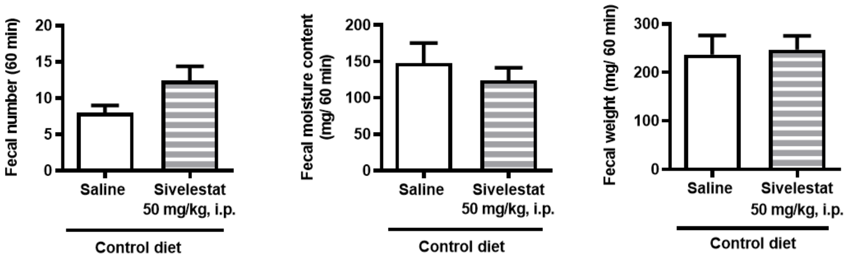

Supplementary Figure S3. Effect of sivelestat (50 mg/kg, i.p.) on the number of fecal pellets, total fecal weight, and fecal water content in the control diet-fed mice (p = 0.0567, 0.4932, and 0.8418, respectively, n = 7–8 group). Student's *t*-test. Data are presented as mean ± SEM for each group.

Uncropped western blot images

The band images at the bottom of each figure in the main text were cropped from these western blot images. LAS 4010 (GE Healthcare) was used to visualize them. In Figures S4 to S7, the lanes indicated with the thick arrows were used for the cropped images in each of the figures. Furthermore, the lanes indicated with the thin arrows are related to quantification.

Supplementary Figure S4.

In Supplementary Figure S4 (for Fig. 1d in the main text), the control diet-fed group is shown in Nos. 1, 3, 5, 7, 9, 11, 13, and 15, and the powder diet-fed group is shown in Nos. 2, 4, 6, 8, 10, 12, 14, and 16, respectively. In AQP4, the western blot images were divided into two parts. However, the samples derive from the same experiment and the experiments were processed in parallel.

AQP4 (48 kDa) (1/2) / Figure 1d

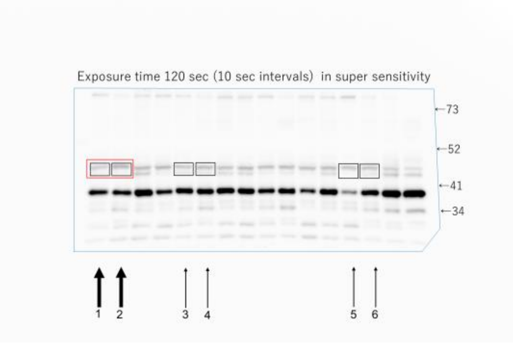

GAPDH (37 kDa) (1/2) / Figure 1d

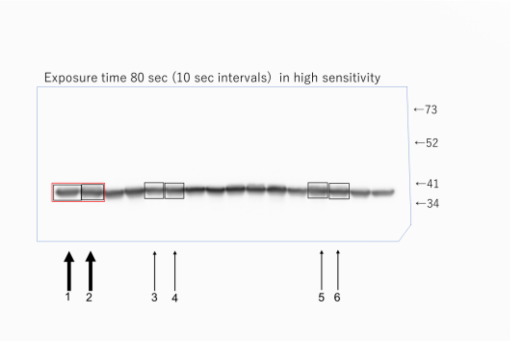

AQP4 (48 kDa) (2/2) / Figure 1d

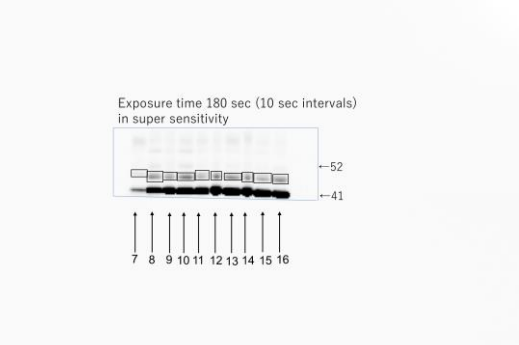

GAPDH (37 kDa) (2/2) / Figure 1d

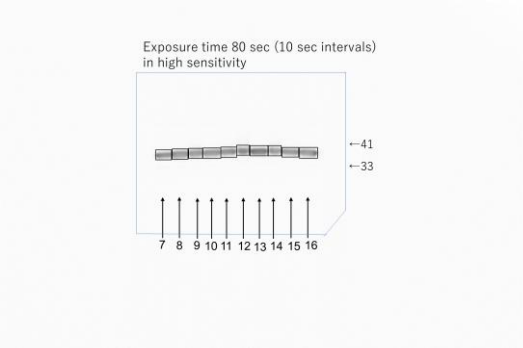

nNOS (160 kDa)/ Figure 1d

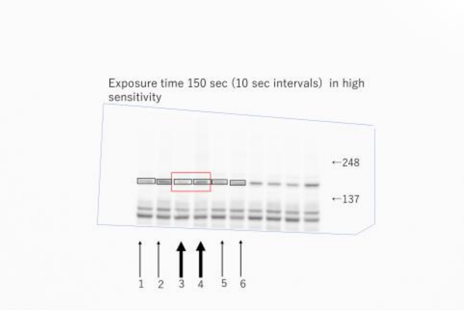

GAPDH (37 kDa)/ Figure 1d

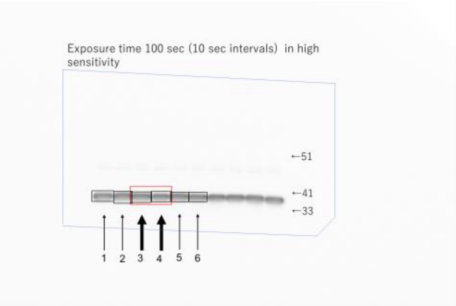

Supplementary Figure S5.

In Supplementary Figure S5 (for Fig. 5c in the main text), the control diet group is shown in Nos.

1, 3, 5, 7, 9, 11, 13, and 15, and the powder diet-fed group is shown in Nos. 2, 4, 6, 8, 10, 12, 14, and 16, respectively. The western blot images were divided into two parts. However, the samples derive from the same experiment and the experiments were processed in parallel.

Ly-6G (25 kDa) (1/2) / Figure 5c

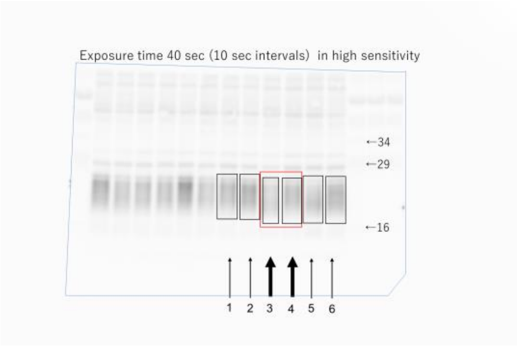

GAPDH (37 kDa) (1/2) / Figure 5c

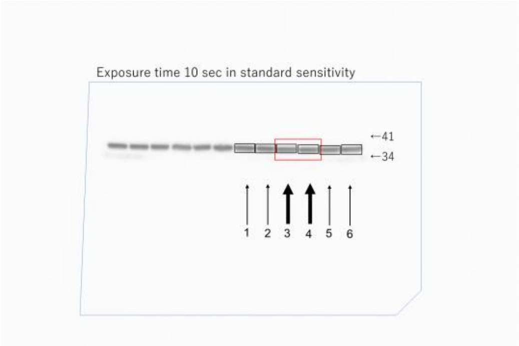

Ly-6G (25 kDa) (2/2) / Figure 5c

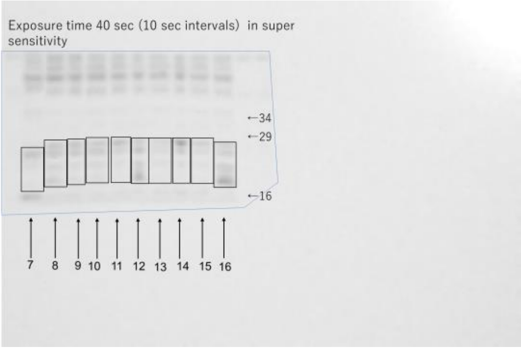

GAPDH (37 kDa) (2/2) / Figure 5c

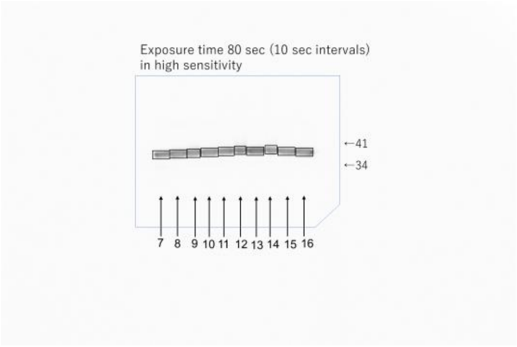

Supplementary Figure S6.

In Supplementary Figure S6 (for Fig. 6c in the main text), the vehicle-treated group is shown in Nos. 1, 3, and 5, and the sivelestat-treated group is shown in Nos. 2, 4, and 6, respectively.

Ly-6G (25 kDa)/ Figure 6c

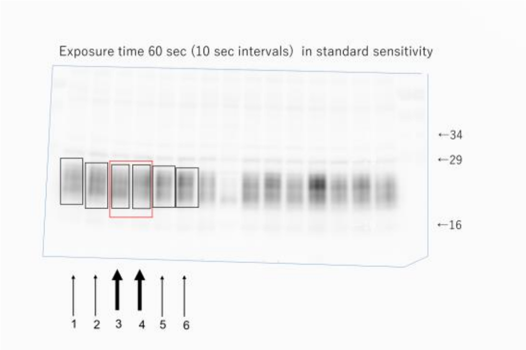

GAPDH (37 kDa)/ Figure 6c

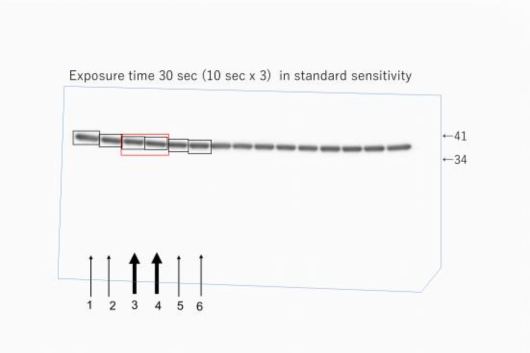

AQP4 (48 kDa) / Figure 6c

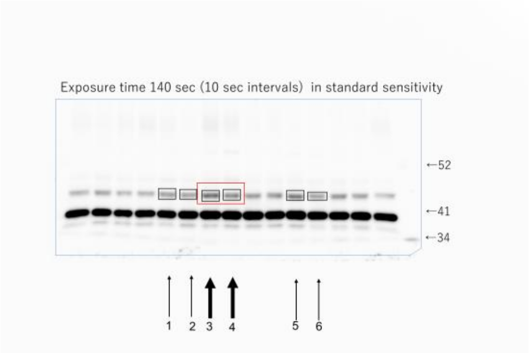

GAPDH (37 kDa)/ Figure 6c

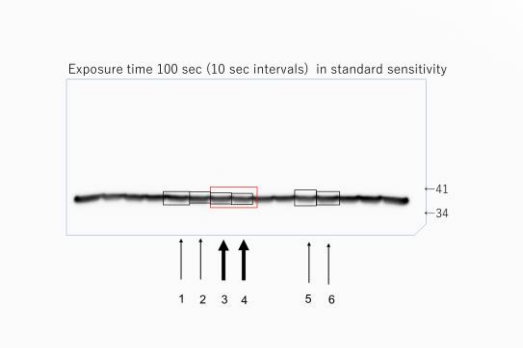

nNOS (160 kDa)/ Figure 6c

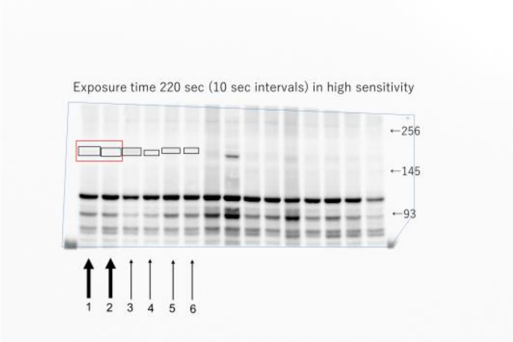

GAPDH (37 kDa)/ Figure 6c

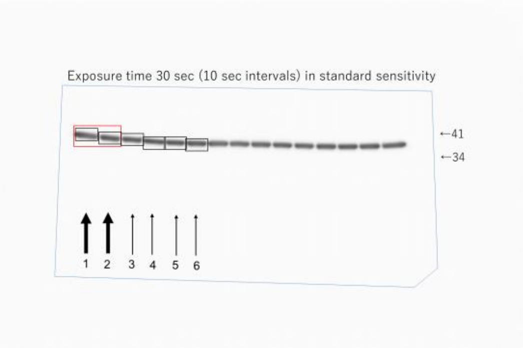

Supplementary Figure S7.

In Supplementary Figure S7 (for Fig. 7b in the main text), the R+G- group is shown in Nos. 1, 3,

and 5, and the R+G+ group is shown in Nos. 2, 4, and 6, respectively.

Ly-6G (25 kDa)/ Figure 7b

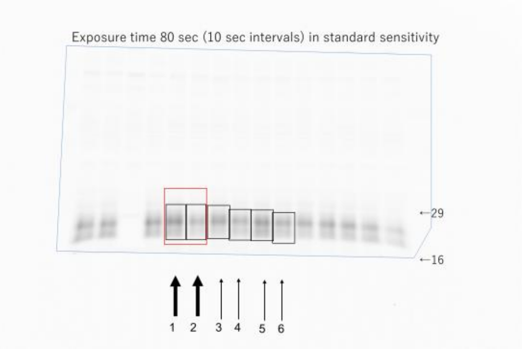

GAPDH (37 kDa) / Figure 7b

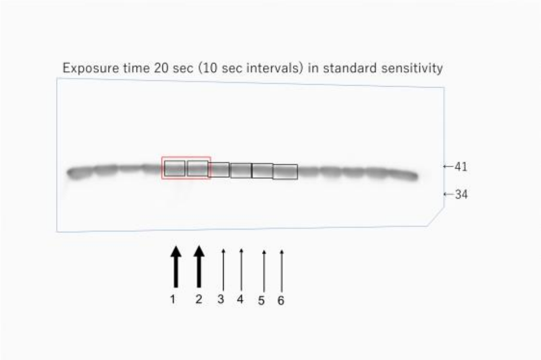

AQP4 (48 kDa) / Figure 7b

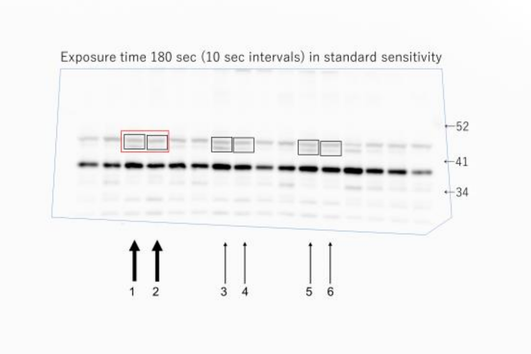

GAPDH (37 kDa)/ Figure 7b

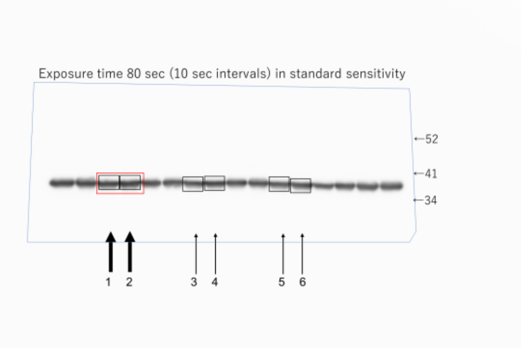

nNOS (160 kDa) / Figure 7b

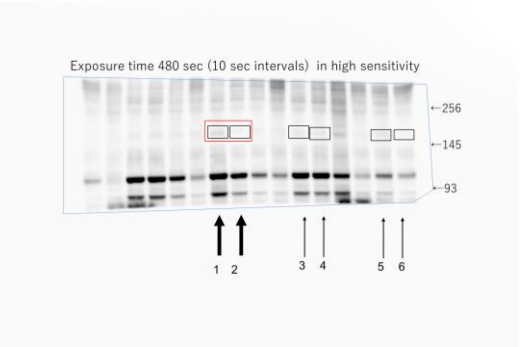

GAPDH (37 kDa) / Figure 7b

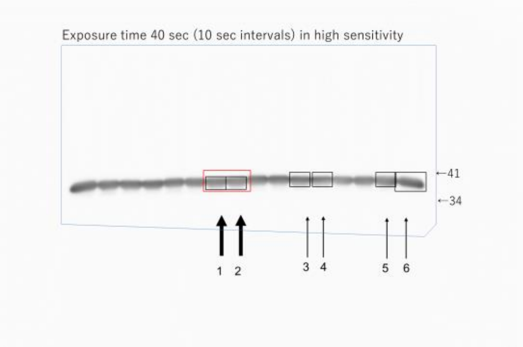

Supplement: Supplementary file 1 — Supplementary Information. [file 41598_2022_18095_MOESM1_ESM.pdf]
